# A realistic 2D multi-offset, multi-frequency synthetic GPR data set as a benchmark for testing new algorithms

**DOI:** 10.1038/s41597-024-04300-1

**Published:** 2025-02-06

**Authors:** G. Roncoroni, P. Koyan, E. Forte, J. Tronicke, M. Pipan

**Affiliations:** 1 University of Trieste, Department of Mathematics, Informatics and Geosciences, Trieste, Italy; 2 University of Potsdam, Institute of Geosciences, Potsdam, Germany

**Keywords:** Geophysics, Scientific data

## Abstract

We present a 2D multi-offset, multi-frequency synthetic GPR data set specifically designed to evaluate and test processing, analysis and inversion techniques. The data set replicates realistic subsurface conditions at four sections separated by 2 m. We modeled four multi-offset GPR profiles at 50, 100 and 200 MHz frequencies using realistic wavelets. The data set provides a robust framework for validating advanced GPR algorithms and techniques such as pre-stack depth migration, amplitude versus offset analysis and full waveform inversion. Extensive technical validation ensures data reproducibility and affordability. The standardized, realistic synthetic data set can be used as a reliable benchmark for developing and testing new algorithms and methods, thereby advancing the understanding of subsurface imaging and real-world data interpretation.

## Background & Summary

Ground-penetrating radar (GPR) is a non-invasive geophysical method widely used for subsurface imaging in various fields such as geology, archaeology, environmental sciences, and civil engineering^1^. Multi-offset (MO) GPR, a technique that involves deploying multiple receivers along a transect to observe various wave types, has gained significant attention due to its ability to enhance subsurface imaging compared to traditional common-offset (CO) applications^2–4^. Specifically, MO GPR can assure that each subsurface point is imaged by multiple wavefronts, while in the commonly employed CO data collection each point in the subsurface is only sampled by a single wavefront. Such a technique is usually referred as to “Multi-fold” (MF) and was originally applied in reflection seismic, contributing to an exponential growth soon after the digital recording revolution. MO GPR offers benefits such as detailed estimates of subsurface electromagnetic wave velocity fields, improved signal-to-noise ratio in reflection sections, and the potential to adapt advanced seismic processing schemes for GPR data^5^. By combining MO data with advanced processing and analysis techniques like pre-stack depth migration^6^ or amplitude-versus-offset (AVO) analysis^7^, it becomes possible to reduce noise, estimate 2D or 3D velocity models, and enhance the quality of subsurface images and estimates of physical parameters^8^.

Quantitative analysis and inversion of MO GPR surveys involve estimating subsurface properties such as moisture content, soil water content, or hydraulic conductivity. Recent advancements in MO GPR have focused on quantitative off-ground approaches, improved resolution through full-waveform inversion, and the utilization of time-lapse measurements to gain new insights into dynamic soil hydrologic processes^9,10^. The application of MO GPR extends beyond traditional GPR studies to areas like agriculture, where it has been used to link horizontal cross-hole variability with root image information for crop root system analysis^11^. Additionally, MO GPR has been employed in environmental studies to monitor dynamic unsaturated flow phenomena and to estimate saturated hydraulic conductivity in sandy soils^12,13^. The technique has also shown promise in detecting and monitoring contaminants like LNAPL (Light Non-Aqueous Phase Liquid) in aquifers^14^. Other successful applications focus on archaeological surveys demonstrating the possibility to obtain improved subsurface imaging, even in highly inhomogeneous subsurface conditions^15^. However, MO GPR is not a standard technique due to its inherent logistical constraints and the very demanding time required to acquire the data.

Full waveform inversion (FWI) is a state-of-the-art technique widely recognized for its effectiveness in imaging subsurface structures and physical parameters using seismic data^16^. Despite its advantages, the implementation of FWI comes with significant challenges that researchers are actively addressing. Recent advancements in the field have seen the application of Convolutional Neural Networks (CNNs) to solve inverse problems in imaging, showing improvements over traditional methods like compressed sensing^17^. Additionally, studies have highlighted the use of numerical simulations as the foundation for GPR inversion techniques, such as FWI, which has shown promise in enhancing resolution compared to conventional methods^18,19^. Researchers have also explored novel approaches like optimizing acquisition setups for cross-hole GPR FWI using checkerboard analysis, which leverages the full recorded signal to improve imaging accuracy^20^. Furthermore, innovative FWI approaches have been developed to estimate parameters like the radius of subsurface cylindrical objects, showcasing the potential of FWI in diverse applications beyond traditional geological studies^21^. Studies have also delved into applications of FWI in assessing concrete properties like chlorides and moisture content, demonstrating the versatility of FWI in material science investigations^22^. Moreover, FWI has been utilized for mapping soil moisture profiles at the field scale, emphasizing the capability of FWI to maximize information retrieval accuracy from GPR data^23^. The integration of FWI with other geophysical data, such as electrical resistivity tomography and reflection seismics^24,25^, presents opportunities for joint inversion approaches that are not commonly explored, but hold promise for future research directions^26^. Additionally, the use of generative adversarial networks in deterministic inversion approaches has been investigated, showcasing the potential of machine learning in enhancing inversion processes^27^.

In recent years, multi-channel GPR equipment has been developed with arrays of antennas, in which each of them can be used either as a transmitter or as a receiver. An array is a noticeable method of increasing the productivity rate by collecting several parallel profiles instead of just one. In this way it is also possible to improve the spatial resolution aiming to collect full-resolution 3D datasets^28^ although not directly exploiting the above-described MF advantages. Examples of 3D GPR surveys with arrays of antennas are reported for archaeological^29–31^, engineering^32^ and road inspection applications^33^.

To collect MF GPR data, several studies with either customary multichannel systems^34^ or combining time-consuming single-channel measurements^2,3^ have been used. Using only one transmitting and one receiving antenna and keeping the azimuth of the antennas constant there are three convenient acquisition geometries exploited to collect MF data sets^4^ Figure Supplementary 1). The easier, but more time-consuming way, is to symmetrically increase the antennas offset keeping their midpoint constant. This acquisition geometry is usually referred to as Common Midpoint Gather (CMP) as in reflection seismics. An alternative way is to move only one antenna away from the other (this geometry is often referred to as Wide Angle Reflection and Refraction – WARR, while in reflection seismic is reported to as Common Shot Gather - CSG). Collecting a series of separated records laterally shifted by a constant distance, it is then possible to combine and sort the data into several CMPs. Another way to obtain MF data, is acquiring several CO profiles along the same path with a different offset each and then sort the data as CMPs. This is the least time-consuming approach, but indeed it is time demanding since a maximum subsurface folding n can be obtained by subsequentially collecting n separated CO profiles, each of them with a different offset. Further details about the different acquisition geometries of MO GPR data can be found in^4^.

Using the last approach^15^, compared CO and MO results for archaeological applications, while^35^ used reflection tomography to estimate water content variations. A multi-channel system with four receiving antennas is reported by^36,37^ to estimate soil porosity and water content using different offsets.

Even though these approaches are promising, all the described strategies have some disturbances because combing single-channel measurements, despite the used acquisition geometry is time consuming^4,38^ and the accuracy depends on the actual location of the antennas on the ground, often requiring smoothing of scattered coordinates and data binning^37^.

A few years ago, a multi-channel and MO equipment (WARR machine or SPIDAR) was tested and launched on the market by Sensors & Software (Mississauga, ON, Canada), allowing measuring with up to eight channels connected with different antennas offsets (almost) simultaneously^39,40^. This new system is specifically developed to make it possible to collect MO GPR data with up to seven different offsets (ranging from 0.25 m to 1.75 m) at the same speed as one single-channel (constant offset) data set. Such a new instrument has been used for different purposes including soil characterization^41^ and improved subsurface velocity analysis^42^.

Despite the previously cited examples, nowadays GPR data sets are mainly acquired using CO geometries and antenna arrays are usually exploited in order to reduce the data acquisition time, increase the spatial resolution, and obtain 3D full-resolution data sets.

It is therefore crucial to have MO (and therefore MF) data with a large enough offset range (depending also on the frequency of the antenna used) and high spatial coverage to properly test the performance of GPR processing, analysis, and inversion algorithms.

In reflection seismics, there is a well-known synthetic data set called “Marmousi” which has been used for more than 35 years as an industry standard and benchmark data set to test, evaluate, compare, and implement advanced processing and inversion techniques. The Marmousi model^43,44^ was created in 1988 mimicking the geometries and the physical parameters of real seismic data of the Kwanza (a.k.a. Quanza) Basin (Angola). Since its original implementation the model was geometrically extended and modified from acoustic to fully elastic, the latter being usually referred to as Marmousi2^45^ as a consequence of the advancement in computer hardware capabilities and new algorithms implementation. More recently, a 3D version of the model was made available for the scientific community^46^ allowing for simulating even complex 3D seismic data sets using various acquisition geometries.

A similar standard does not exist for GPR and, up to now, there are no MO synthetic but realistic GPR data sets made available to the scientific community to test new algorithms and procedures. It limits the reproducibility of the results and makes difficult to understand the subjectivity in choosing parameters and defining flows. To overcome these limitations, we simulate, present, and make publicly available a MO and multi-frequency data set across the model made available by^47^ for which the same authors already provided a 3D single frequency CO data set^48^.

## Methods

**Fig. 1:**
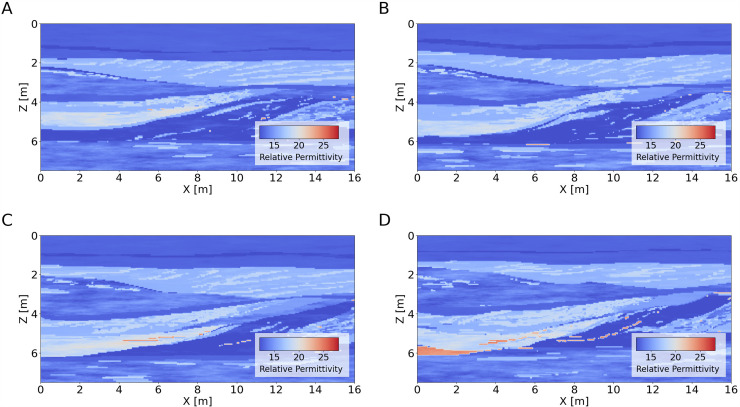
Relative permittivity [adimensional] along the 4 selected sections from the Herten 3D model. (**A**) y = 2 m; (**B**) y = 4 m; (**C**) y = 6 m: (**D**) y = 8 m.

The sedimentary model used in the 3D modeling of GPR data bases on a high-resolution hydrofacies data set obtained from an aquifer-analog study within fluvio-glacial deposits^49^. The model represents a gravel quarry near the village of Herten in SW-Germany, where sand and gravel sequences formed in a braided-river regime characterize the near surface. The data set includes detailed hydrogeological properties and their spatial distributions within the quarry, covering an area of 16 × 10 m (*x*x*y*) and a depth range (*z*) of 7 m with a resolution of 0.05 m^50^. From this 3D model, we select 4 sections at 2 m, 4 m, 6 m, and 8 m along the *y* dimension, as depicted in Fig. 1 in terms of relative permittivity assuming a fresh-water saturated scenario.

**Fig. 2:**
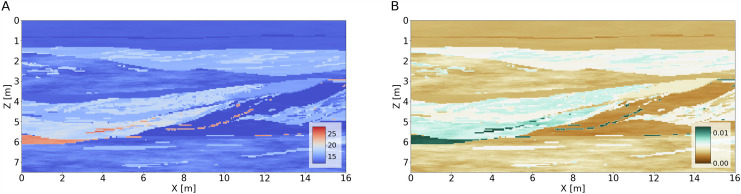
Relative permittivity [adimensional] (**A**) and conductivity [S/m] (**B**) for the section at y = 8 m as depicted in Fig. 1D.

The sedimentary model exhibits a variety of realistic features at different spatial scales, including thin interfaces and dipping layer sequences with varying electrical parameter contrasts (Fig. 2). These features make the model a challenging yet ideal target for testing and evaluating novel 2D GPR processing and inversion methods, for example FWI, migration or deconvolution algorithms.

To perform forward modeling (i.e., to simulate synthetic GPR data), we use gprMax v.3.1.7^51,52^, an open-source electromagnetic modeling software (https://github.com/gprmax/gprMax) specifically developed for simulating GPR data using the Finite-Difference Time-Domain (FDTD) numerical method^53^. By utilizing the propagation physics of electromagnetic waves and the FDTD method, gprMax enables accurate simulation of GPR signals, making it valuable for investigating signal processing approaches and enhancing interpretation skills^54,55^. One of the notable strengths of gprMax is its capability to simulate real-world GPR scenarios, providing users with insights into expected outcomes during surveys and aiding in the enhancement of signal processing and interpretation capabilities^56^. Additionally, gprMax is fully parallelized, enabling it to leverage multiple CPUs and GPUs for efficient and high-performance simulations^57^. The simulation is performed using the parameters reported in Table 1.

**Table 1:** Simulation parameters for the synthetic GPR data for the three different central frequencies equal to 50, 100 and 200 MHz, respectively.

Central frequency *f* [MHz]	Timestep *dt* [ns]	Recording time *t* [ns]	Model discretization *d*_*xz*_ [m]	Source/receiver position *x*_*src*_*/x*_*rec*_ [m]	Source/receiver position *y*_*src*_*/y*_*rec*_ [m]	Source/receiver position *z*_*src/*_*z*_*rec*_ [m]
50	0.116	500	0.05	0:0.1:16	2:2:8	0
100	0.058		0.025			
200	0.029		0.0125			

**Fig. 3:**
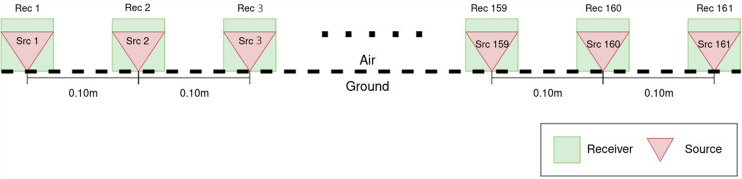
Sketch of the acquisition geometry used for all profiles and each frequency. The 161 Receivers have fixed locations, while the 161 source locations (shot points) are moved by steps of 0.1 m from position 1 to position 161.

The acquisition geometry is depicted in Fig. 3. For each of the four sections selected within the model, we simulate 161 shots (i.e., one source every 0.1 m from 0 m to 16 m) and, in each case, record the EM field using 161 receivers (i.e., one receiver every 0.1 m from 0 m to 16 m). A detailed sketch of the model used for the forward modeling including the air layer and PML region can be found in Figure Supplementary 2.

**Fig. 4:**
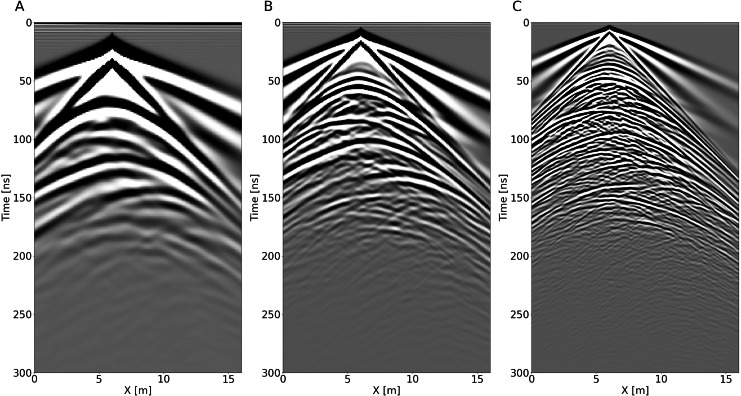
Comparison of three CSG simulated at x_src_ = 6 m with 50 MHz (**A**), 100 MHz (**B**), and 200 MHz (**C**) central frequencies.

We generate 161 CSG for each of the four sections and for each of the three frequencies. We therefore provide a data set of 12 MO and multi-frequency GPR profiles: we simulate four 2 m separated GPR profiles for 50, 100 and 200 MHz central frequency Ricker wavelets. These wavelets and their spectra are shown in Figure Supplementary 3. An example CSG for each frequency is shown in Fig. 4. A more detailed example of four CSG for each frequency is shown in Figures Supplementary 4–6, for the 50, 100 and 200 MHz, respectively.

In order to demonstrate the differences between each profile within the model, the CSG at *x*_*src*_ = 6 m is shown for each of the four sections in Figures Supplementary 7–9, for the 50, 100 and 200 MHz, respectively.

Comparing the CSG at different frequencies (Fig. 4), we observe a substantial increase in resolution with increasing frequency, making the data set an ideal test case for frequency-based inversion, such as frequency FWI, frequency merging^58^, or data deconvolution.

## Data Records

The dataset supporting this study is available on Figshare^59,60^. It includes synthetic multi-offset and multi-frequency ground-penetrating radar (GPR) data, stored in SEG-Y format, alongside the scripts and resources necessary for processing and validation.

The GPR data is organized into subdirectories by frequency (50 MHz, 100 MHz, and 200 MHz), with each directory containing four profiles corresponding to the model sections at y = 2 m, y = 4 m, y = 6 m, and y = 8 m, *line0* to *line3*, respectively. Further details about the organization of the files and the structure of the dataset can on the data repository^59,60^. Each SEG-Y file is accompanied by header information specifying acquisition parameters, as outlined in the Usage Notes section.

Jupyter notebooks provided in^59,60^ demonstrate how to read SEG-Y files, visualize the profiles, and verify the acquisition geometry using python. The required computational environment is defined in conda environment file: *env.yml*.

## Technical Validation

### Data stability

**Fig. 5:**
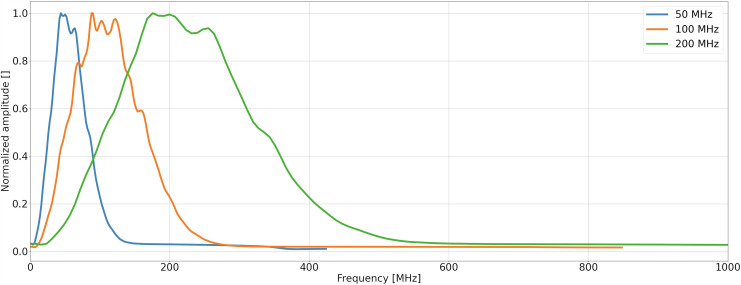
Mean amplitude spectra among each frequency data set, each normalized to 1. (**A**) 50 MHz, (**B**) 100 MHz, (**C**) 200 MHz. The stability of the simulation is evident due to the absence of both high and low spurious frequency components.

In order to illustrate the effectiveness of the forward modelling, we show the mean frequency spectrum for each frequency (Fig. 5). It is apparent that the spectra are centered around the expected central frequencies, with no spurious effects at either low or high frequencies edges (as they would be expected in case of data instability due to wrong model parametrization).

**Fig. 6:**
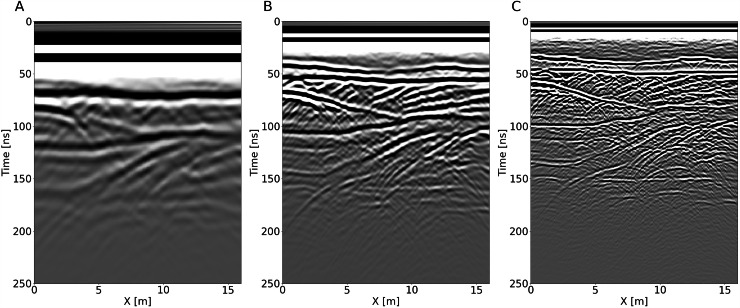
Example zero-offset profile with central frequencies of 50 MHz (**A**), 100 MHz (**B**), and 200 MHz (**C**). Due to the different shapes of the used source wavelets (see Figure Supplementary 3) the first arrivals (air waves) have slightly different time shifts.

Furthermore, we compare the zero-offset GPR profiles (i.e., *x*_*src*_ = *x*_*rec*_) for each frequency for the section at y = 2 m (Fig. 6). We observe near-field effects, which are larger for lower frequencies, as expected. These near-field effects occur within the close range of the GPR antenna, where emitted electromagnetic wavefronts have not yet fully developed into far-field radiation patterns. For lower frequencies, such as 50 MHz, the near-field zone is wider, reaching a time length of about 70 ns (Fig. 6A). This extended near-field zone is due to the longer wavelengths associated with lower frequencies, which increase the distance over which the electromagnetic field transitions from the near-field to the far-field. Consequently, this makes not possible to extract the signal from the noise in the shallower portion of the data.

In contrast, higher frequencies, such as 200 MHz, exhibit a much shorter near-field region, not exceeding 20 ns (Fig. 6C). The reduced near-field zone at higher frequencies allows improved separation between the direct wave and the shallowest reflections.

The increasing times of the reflectors with decreasing antenna frequencies in Fig. 6 can be attributed to the time lag of the Ricker wavelet used in the forward modelling, which decreases as the frequency increases. Specifically, the 50 MHz wavelet has a longer time lag compared to the 100 MHz and 200 MHz wavelets, as shown in Figure S9. This results in progressively shallower apparent depths for higher-frequency antennas. A zero-time correction is typically applied to real GPR data in the first steps of the processing flow^1^ and can also be applied to this dataset.

### Data compatibility and geometry validation

SEG-Y data provided in this paper (see Table 2 for headers) are tested to properly work on different commercial and open-source programs originally developed for both reflection seismic: ProMAX (Halliburton), Petrel 17 (Schlumberger), Seisee 2.22 (Dalmorneftegeofizika Geophysical Company), and GPR: Prism 2.70.04 (Radar Systems), ReflexW 9.5.7 (Sandmaier geophysical research).

**Table 2:** Location [byte] of the header information stored in the SEG-Y files.

Header	Start byte	End byte
Trace number	5	9
Trace ID	9	13
Recorder ID	13	17
Shot ID	17	21
Source X [cm]	73	77
Receiver X [cm]	81	85
CMP [cm]	181	185

**Fig. 7:**
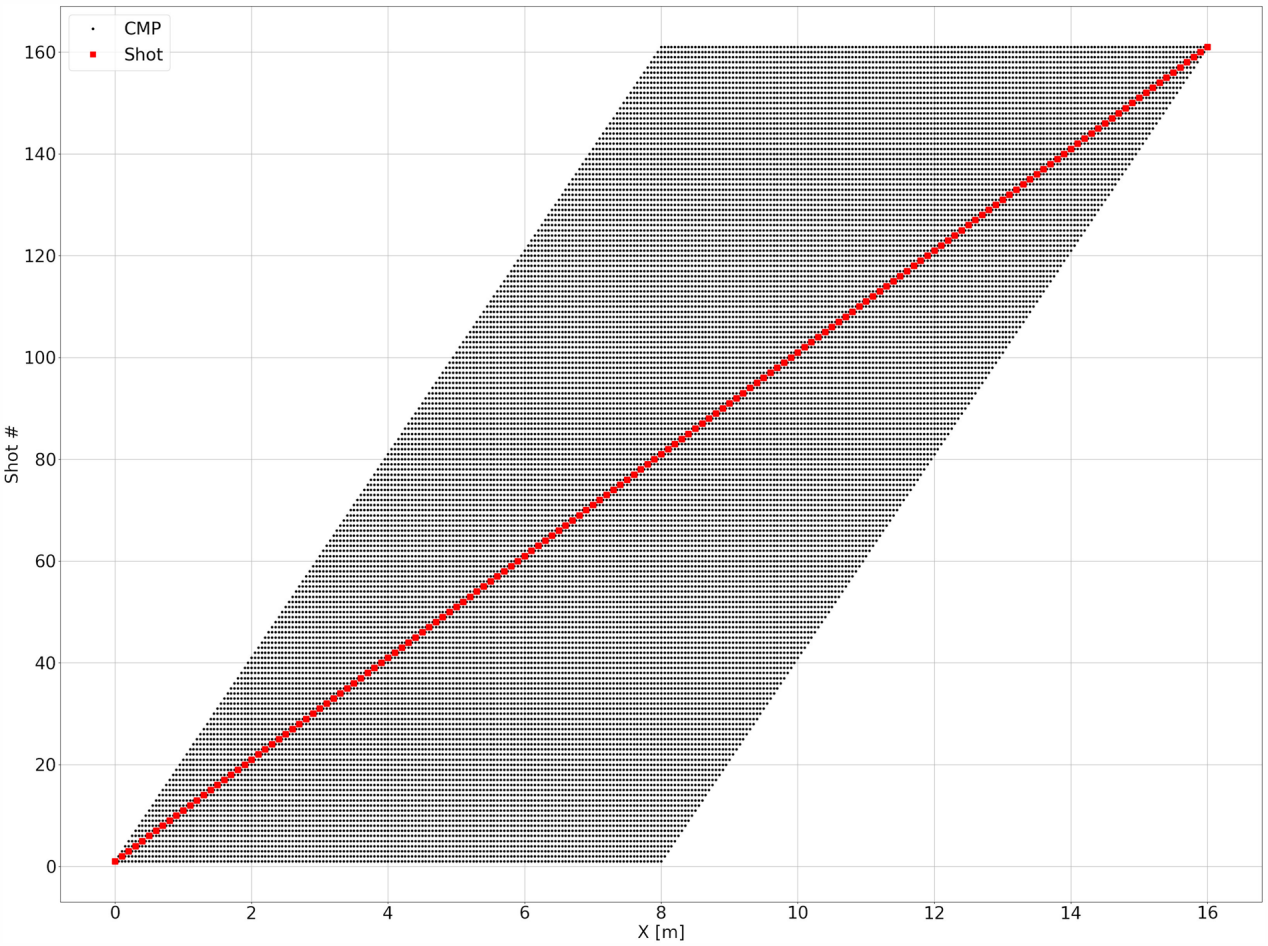
Stacking chart of the acquisition geometry. CMP are plotted as black dots, sources as red dots.

In order to validate the data geometry, we analyze the stacking chart for the entire simulated data set. Figure 7, shows the shot numbers vs their *x* coordinate. The maximum folding (i.e., 161) is correctly reached for the CMP at 8 m.

**Fig. 8:**
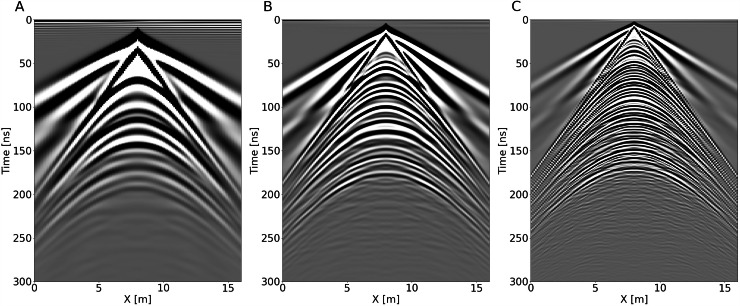
CMP gathers for the central transmitter location (x = 8 m) along the y = 8 m section for central frequencies of 50 MHz (**A**), 100 MHz (**B**), and 200 MHz (**C**).

In order to further validate the acquisition geometry, we compare three CMP at *x* = 8 m, that is, the full-folding case (Fig. 8).

## Usage Notes

An example python code to read the header file can be found at^59^ and in https://github.com/Giacomo-Roncoroni/MO-GPR_data.

## Supplementary information

Supplementary information

## Data Availability

Codes for data validation and exemplary gprMax input files are reported in^59^ and in https://github.com/Giacomo-Roncoroni/MO-GPR_data.
