# Supplementary material for: A realistic 2D multi-offset, multi-frequency synthetic GPR data set as a benchmark for testing new algorithms
Source: Sci Data. 2025 Feb 6;12:221. doi: 10.1038/s41597-024-04300-1 (PMC11802766; doi:10.1038/s41597-024-04300-1)
Supplement: Supplementary file 1 — Supplementary information [file 41597_2024_4300_MOESM1_ESM.docx]

A realistic 2D multi-offset, multi-frequency synthetic GPR data set as a benchmark for testing new algorithms

Roncoroni^1^, P. Koyan^2^, E. Forte^1^, J. Tronicke^2^, M. Pipan^1^

^1^University of Trieste, MIGE, Trieste, Italy

^2^University of Potsdam, Institute of Geosciences, Potsdam, Germany

**Supplementary Materials**

**
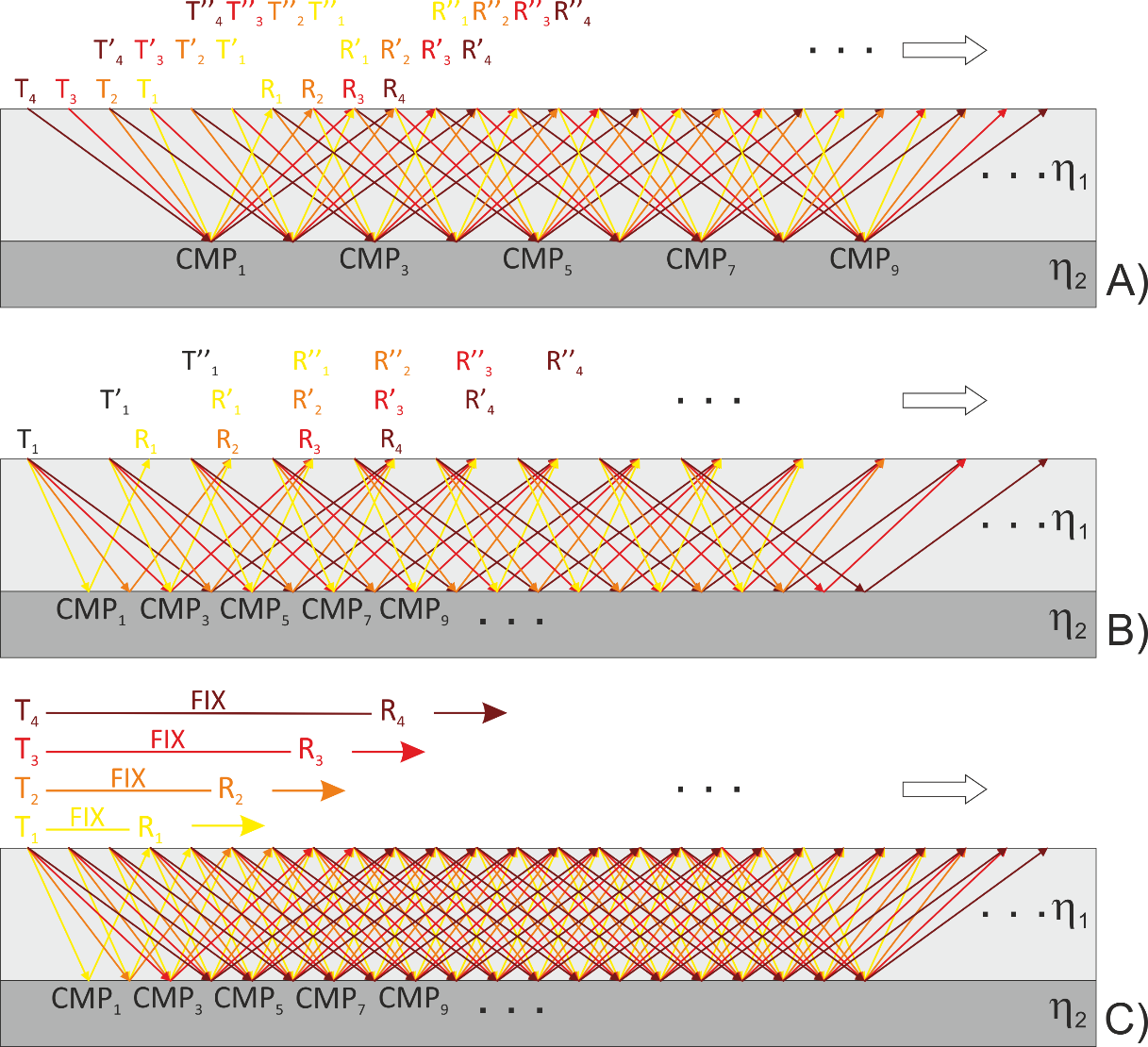
**

***Figure Supplementary 1****: MO acquisition schemes for single channel GPR instruments: A) series of CMP; B) series of CSG; C) series of CO profiles. T and R are the transmit and receive antenna positions, respectively. Different colors correspond to different offsets.*

**
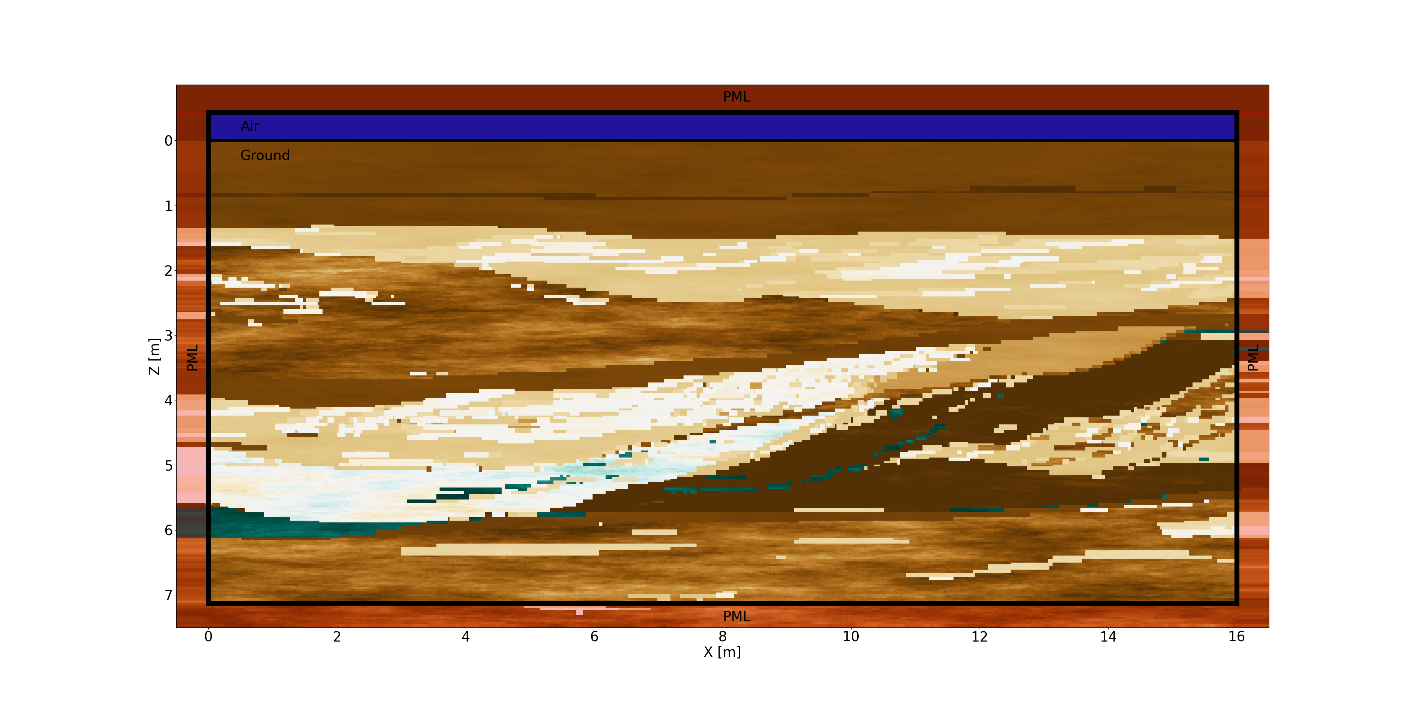
**

***Figure Supplementary 2****: Sketch of the acquisition setup: PML regions are in red, with the area with x<0m and x>16m horizontally padded. In blue the Air and the antennas is located in air at the interface with ground, i.e. last cell of air before the ground starts.*


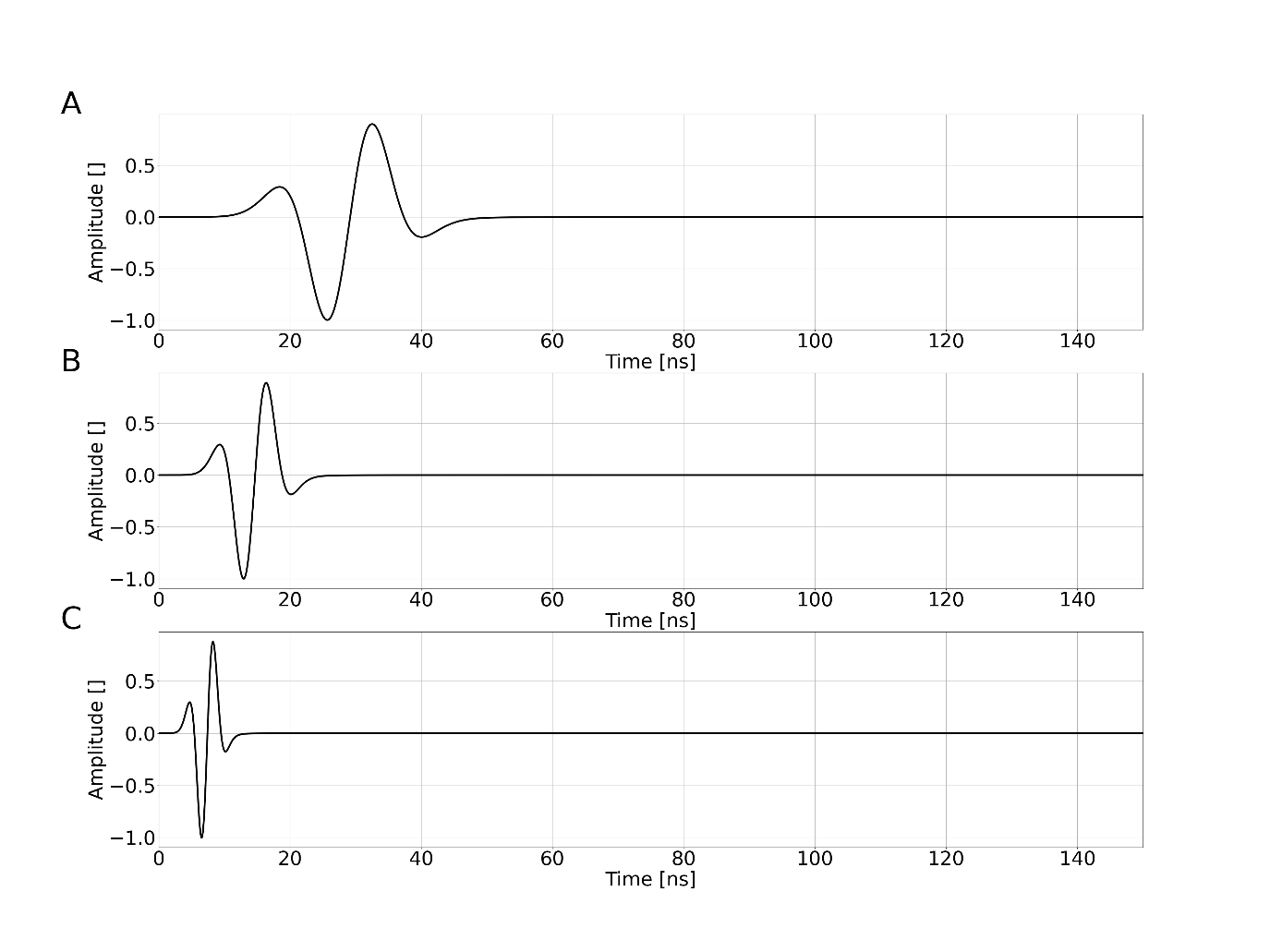


***Figure* Supplementary 3***: Plot of the waveforms recorded in air of the Hertzian Dipole used for 50Mhz (A), 100MHz (B) and 200MHz (C).*

**
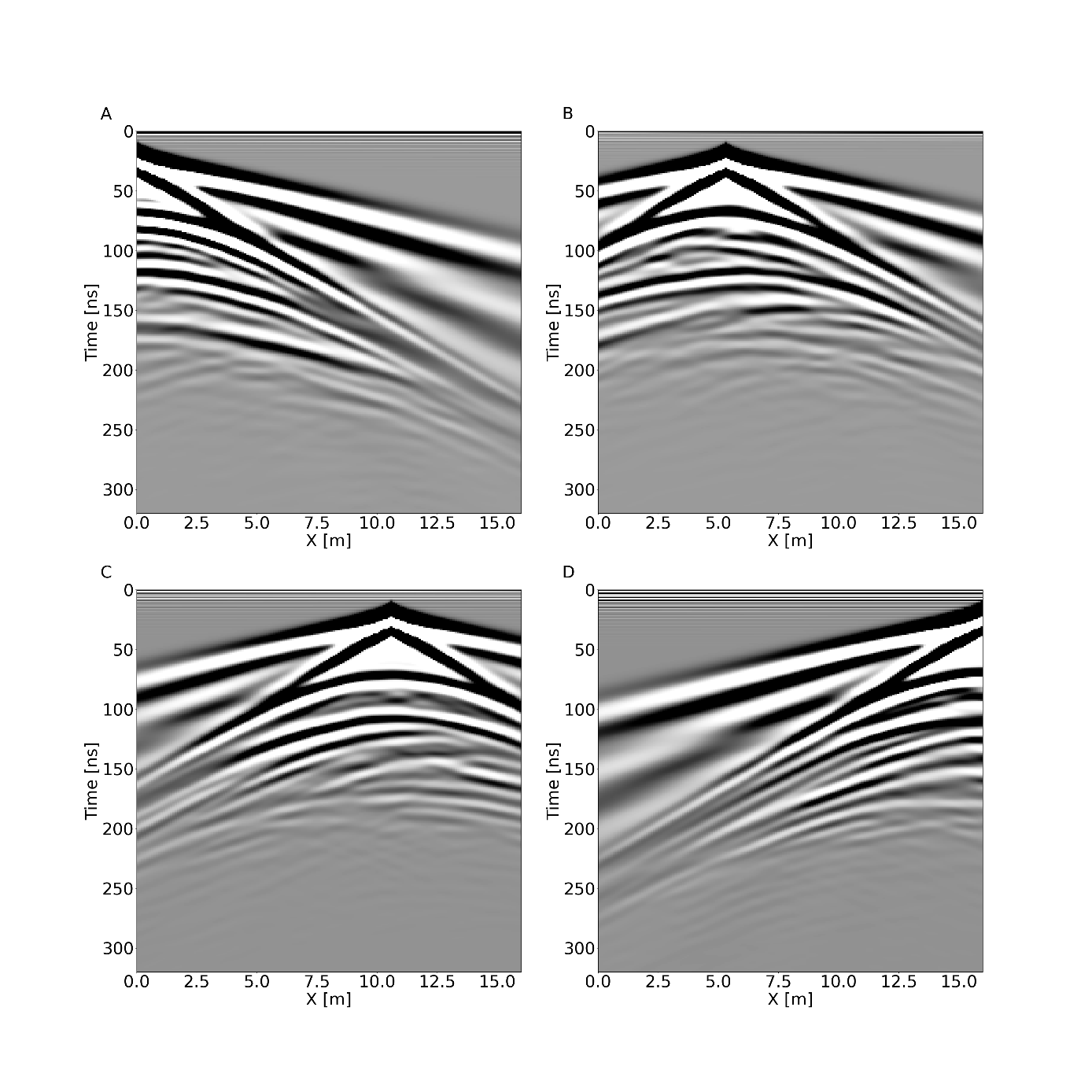
**

***Figure Supplementary 4****: Four CSG of Line 8m from the 50MHz dataset generated at: 0m (A), 5m (B), 10m(C) and 16m (D).*

**
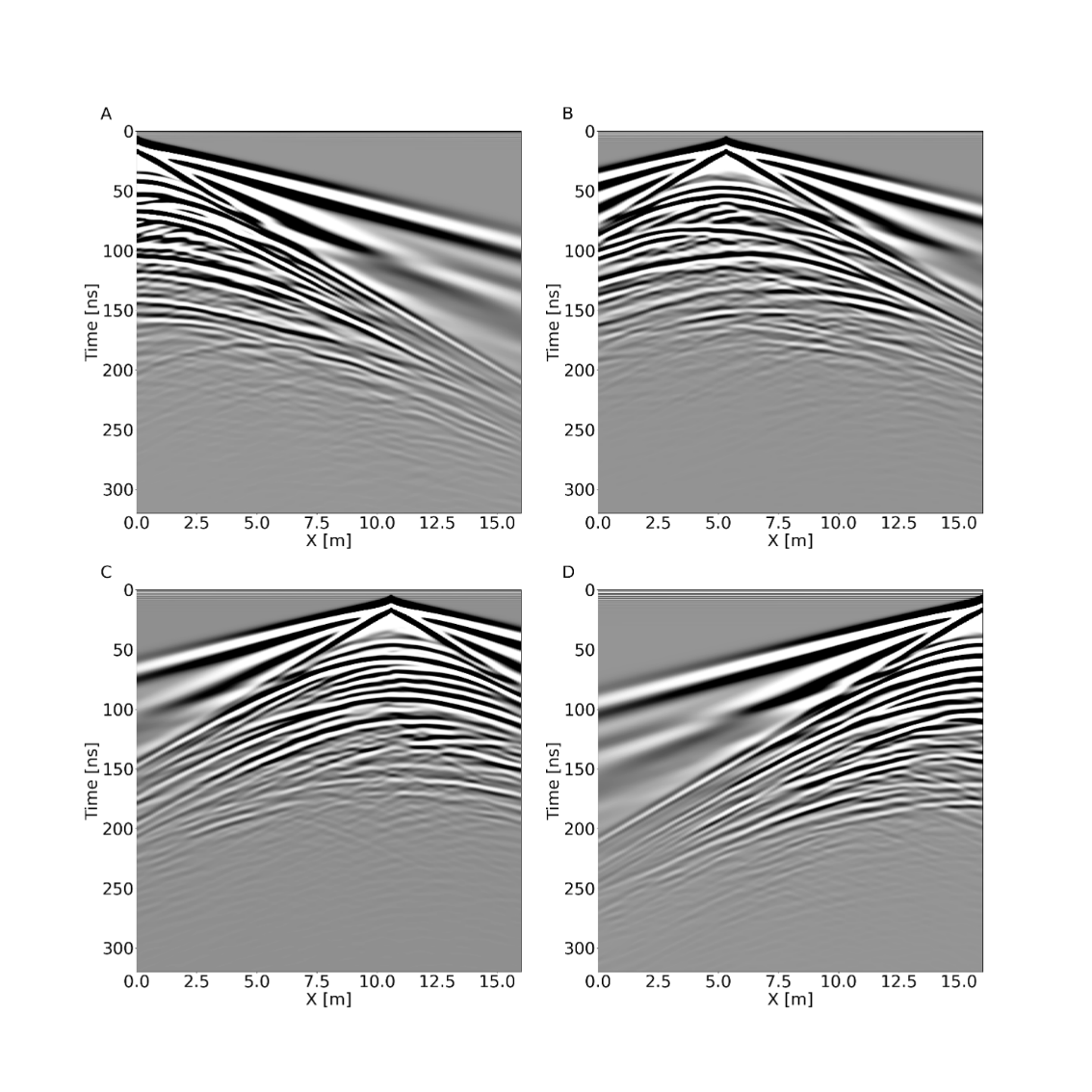
**

***Figure Supplementary 5****: Four CSG of Line 8m from the 100MHz dataset generated at: 0m (A), 5m (B), 10m(C) and 16m (D).*

**
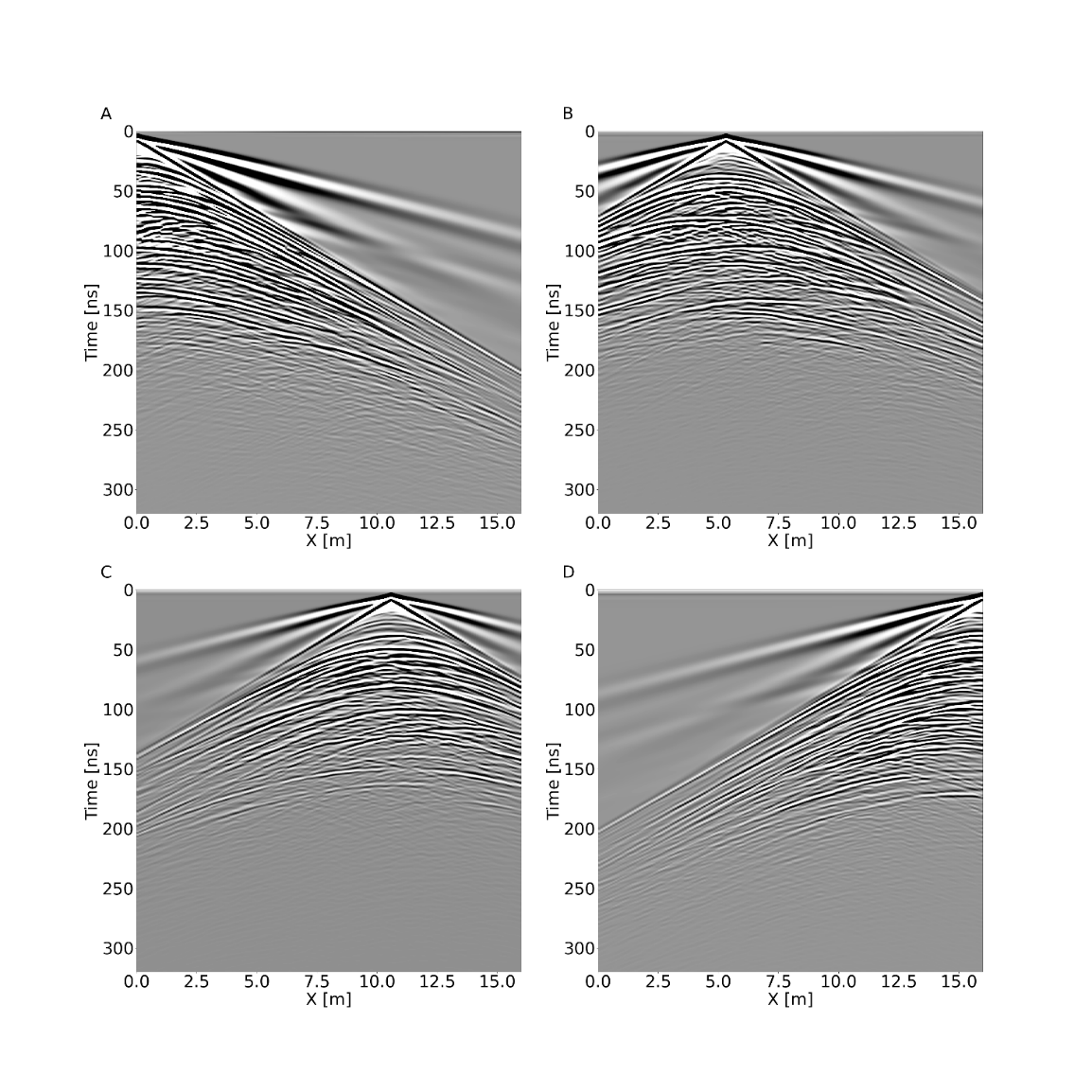
**

***Figure Supplementary 6****: Four CSG of Line 8m from the 200MHz dataset generated at: 0m (A), 5m (B), 10m(C) and 16m (D).*

**
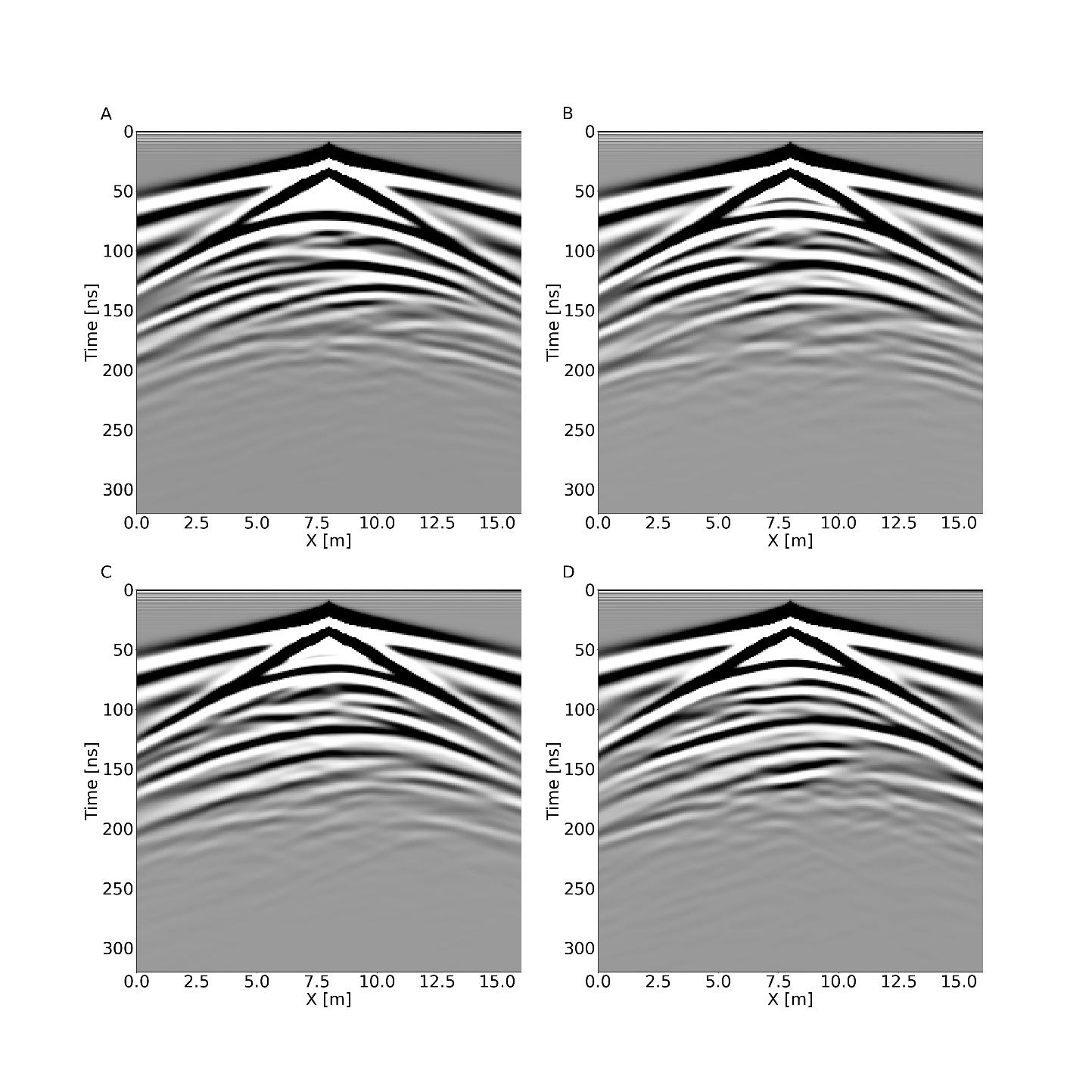
**

***Figure Supplementary 7****: Four CSG at 50MHz with source position at 7.5m for each of the 4 2D model: A is from Line 2m, B is from Line 4m, C is from Line 6m and D is from line 8m.*

**
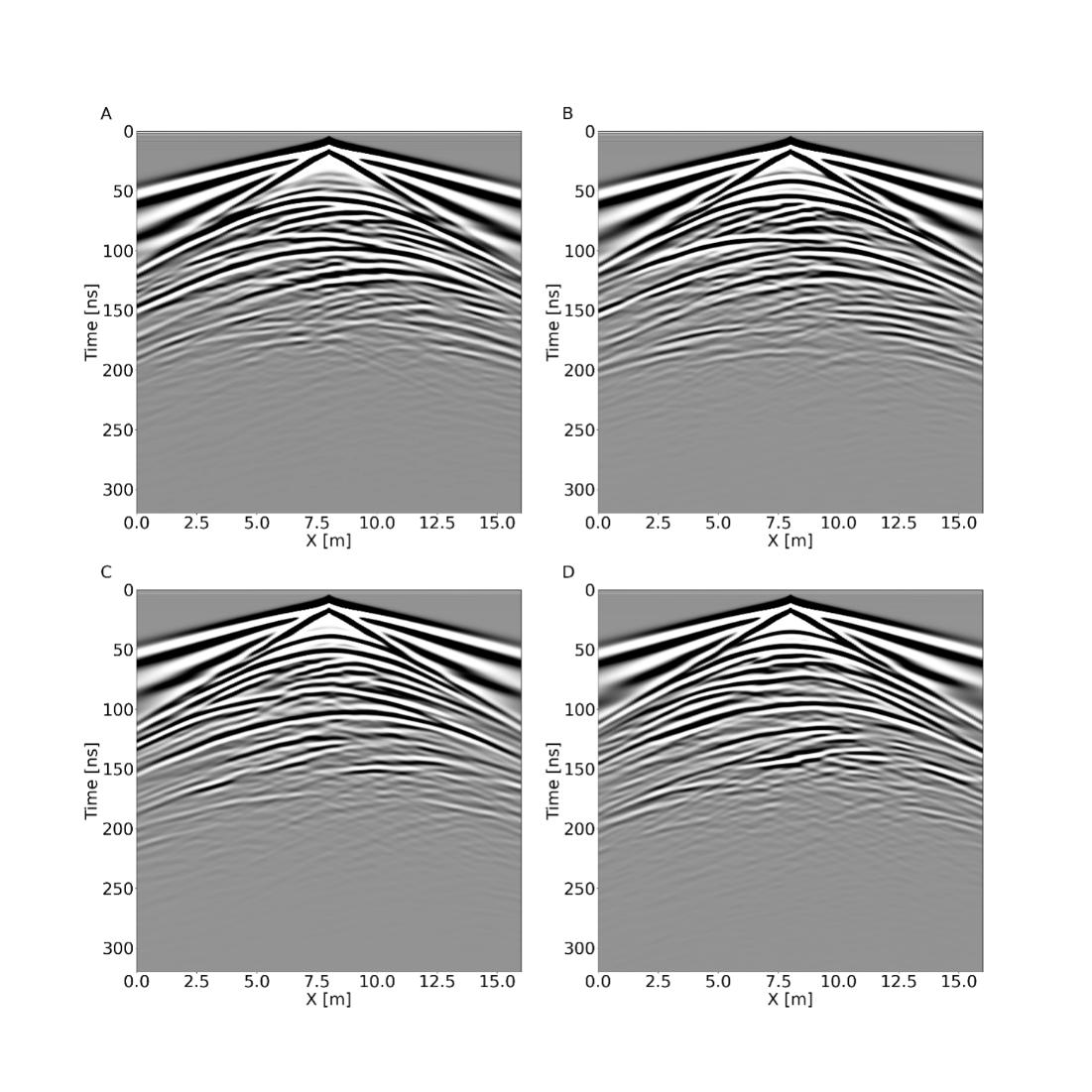
**

***Figure Supplementary 8****: Four CSG at 100MHz with source position at 7.5m for each of the 4 2D model: A is from Line 2m, B is from Line 4m, C is from Line 6m and D is from line 8m.*


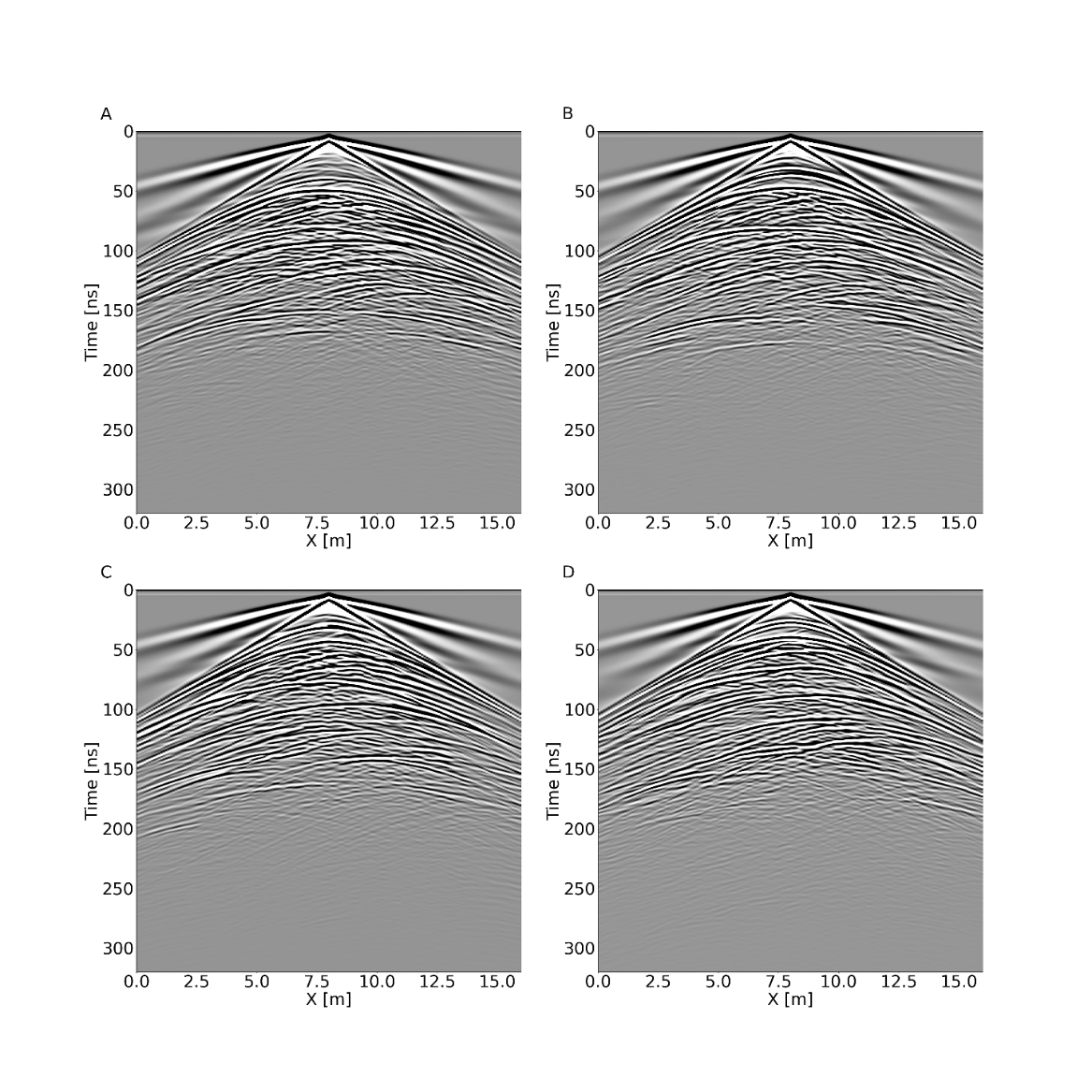


***Figure Supplementary 9****: Four CSG at 200MHz with source position at 7.5m for each of the 4 2D model: A is from Line 2m, B is from Line 4m, C is from Line 6m and D is from line 8m.*
